# 

**Published:** 1896-12-05

**Authors:** 


### EARLY EDITION.

**Notices of Births, Deaths, and Marriages** are inserted in this column at a charge of 2s. 6d. for an announcement not exceeding four lines.

**NOTICE TO CORRESPONDENTS.**

All MS., Letters, Books for Review, and other matters intended for the Editor should be addressed The Editor, "The Hospital," The Hospital Building, 28 & 29, Southampton Street, Strand, W.C.

The Editor cannot undertake to return rejected MS., even when accompanied by stamped directed envelope.

**Notice to Advertisers and Subscribers.**

All Advertisements, Orders for Copies of The Hospital, and Business Communications should be addressed to **THE MANAGER, (Not to The Editor),** The Hospital, The Hospital Building, 28 & 29, Southampton Street, Strand, London, W.C.

The Cost of Subscription, post free, is as follows:—Per week, 2½d.; per quarter, 2s. 8d.; per half-year, 5s. 3d.; per annum, 10s. 6d. Foreign:—Per annum, 15s. 2d.; payable, in all cases, in advance.

**NOTICE.—Vol. XX. of "The Hospital" (April to September, 1896), in handsome cloth binding, is now on sale at the Office of this Journal, price 7s. 6d. Cases for binding the Numbers contained in this Volume 1s. 6d. Index to Vol. XX., 2½d., post free. The Monthly Part for December is now ready, Price 6d., by post 8d.**

### The Student of Medicine.

### APPOINTMENTS.

F. S. Barnett, M.R.C.S.Eng., appointed Medical Officer for the Lewisham District of the Lewisham Union. R. A. Beaver, M.B., L.R.C.P.Lond., M.R.C.S., appointed Medical Officer for the Hinton St. Mary District of the Sturminster Union. A. H. Boucher, M.D.Edin., D.P.H.Camb., appointed *pro tem.* Medical Officer of Health to the Hertfordshire Combined District. Sir W. H. Broadbent, Bart., M.D.Lond., appointed Consulting Physician to the Victoria Hospital for Sick Children, Chelsea. T. T. Brunyate, M.A., M.D., appointed Honorary Surgeon to St. Bartholomew's Hospital, Rochester. A. E. Giles, M.D.Lond., B.Sc., F.R.C.S.Edin., appointed Surgeon to the Out-patients at the Chelsea Hospital for Women. W. J. Greer, F.R.C.S. and D.P.H.Irel., appointed Certifying Factory Surgeon at Newport, Mon. A. Hind, L.R.C.S.Edin., L.M., L.S.A., appointed Medical Officer for the Chittlehampton District of the South Molton Union. R. Kennedy, M.A., B.Sc , M.B , C.M., appointed Dispensary Surgeon to the Western Infirmary, Glasgow. W. A. Milligan, M.A., M.B., C.M.Aberd., appointed Junior House Surgeon to the Stanley Hospital, Liverpool. J. Morton, M.B., C.M.Glasg., appointed Extra Dispensary Surgeon to the Western Infirmary, Glasgow. W. G. Nash, F.R.C.S., appointed Surgeon to the Bedford General Infirmary. H. W. Newton, M.R.C.S., L.R.C.P., appointed Surgeon H.M. Prison, Chelmsford. A. G. Phear, M.D.Cantab., M.R.C.P.Lond., appointed Assistant Physician and Pathologist to the Metropolitan Hospital. J. F. Saunders, M.R.C.S.Eng., L.S.A., appointed Medical Officer for the Catford District of the Lewisham Union. M. T. Stack, M.B., C.M.Glasg , appointed Assistant House Surgeon to the Taunton and Somerset Hospital. W. F. M. Wallace, M.B., C.M., appointed Medical Officer and Public Vaccinator for the Eastern District of the Parish of Dysart. S. J. Wareham, M.R.C.S.-Eng., L R.C.P.Lond., L.S.A.Lond., appointed House Physician to Charing Cross Hospital. H. L. Webb, M.R.C.S., L.S.A., appointed Medical Officer of Health to the Cheadle Rural District. M. Yearsley, F.R.C.S., appointed House Surgeon to the Royal Ear Hospital, Soho, W. A. A. Young, M.A.Glasg., M B , C.M , appointed Dispensary Surgeon to the Western Infirmary, Glasgow.

### UNIVERSITIES AND COLLEGES.

### University of Edinburgh.

Statistics of Medical Preliminary Examination.—Fifty-three candidates passed the University Preliminary Examination; 7 passed partly with outside exemptions; 82 passed entirely with outside exemptions; 6 were exempted by the M.A. qualification. Thus 148 completed the Preliminary Examination in October, 1896, as against 128 in October, 1895. To these numbers must be added 70 students who formerly completed the Preliminary Examination or are otherwise accounted for, bringing the total number of first-year students up to 218, as appeared in the *British Medical Journal* a fortnight ago. Six women students have matriculated in the Faculty of Medicine for the present session, and there have been recorded in the University books 48 extra-academical graduation students of medicine, making a total of 54 women students of medicine working towards graduation.

University Court.—At the last meeting of the University Court the Right Hon. Lord Balfour of Burleigh, who took his seat as a member of the Court, presided as *ex-officio* President of the meetings of the Court. Intimation was made of a legacy of £1,000 by the late Dr. W. F. Rawdon, York, to the building fund of the University, in which University he graduated as M.D. in the year 1827. On the recommendations of the professors of the respective departments, the Court appointed Mr. J. P. Longstaff to be fourth assistant in Chemistry; A. C. Sturrock, M.B., C.M., to be third assistant in Physiology; and David Waterston, M.B., C.M., to be fourth assistant in Anatomy. The Court had under consideration a letter from the President of the General Council of Medical Education, regarding the disciplinary powers of the Universities over their graduates, and copy of a report on the disciplinary or penal powers of the qualifying medical authorities as approved by the General Medical Council.

### University of Aberdeen.

Veterinary Hygiene.—In connection with the Agricultural Department, which, by means of State grants and contributions from the city of Aberdeen and the County Councils of Aberdeenshire and Banffshire, has lately undergone great development, Mr. J. M'Lauchlan Young, M.R.C.V.S., Dundee, has been appointed Lecturer on Veterinary Hygiene. For several years Mr. Young has been well known as a scientific investigator and lecturer in veterinary science.

### Royal College of Surgeons in Ireland.

Fellowship Examination.—The following gentlemen have passed the primary part of the examination: D. A. Fitzgerald and J. Foley. The following gentlemen having passed the final part of the examination have been admitted Fellows of the College: A. R. Friel, J. Pim, C. Ward.

